# FPGA-based reconfigurable scanning and data acquisition system for scanning electron microscopy

**DOI:** 10.1186/s42649-026-00137-7

**Published:** 2026-05-23

**Authors:** Kyubin Gong, Junseok Kim, Bog G. Kim, Boklae Cho

**Affiliations:** 1 Department of Nano Semiconductor Process and Equipment, Pusan National University, Busan, 46241 South Korea; 2 Department of Physics, Pusan National University, Busan, 46241 South Korea; 3 ModuleSci Co., Ltd., Daejeon, 34054 South Korea

**Keywords:** FPGA, Scanning electron microscopy, Data acquisition, Raster scan, Real-time synchronization, Signal-to-noise ratio, Sub-pixel drift correction, Modulation transfer function

## Abstract

We present an FPGA-based reconfigurable scanning and data acquisition system for scanning electron microscopy (SEM). Built on the Xilinx Artix-7 (XC7A35T), the system integrates dual-channel 14-bit DAC raster scan waveform generation, dual-channel 12-bit ADC signal acquisition with on-chip averaging, and real-time USB 2.0 High-Speed data streaming at up to 40 MB/s. Integration with a commercial SEM (ModuleSci PicoEye-100) produced clearly resolved secondary-electron images, demonstrating stable raster operation in the fast-scan mode used for alignment and focusing. Standard data acquisition was performed at a per-frame acquisition time of 10 s, and a quantitative image-quality benchmark against the instrument’s built-in acquisition channel under this condition, using a grid-hole masking protocol and sub-pixel cross-correlation drift correction (Guizar-Sicairos et al. 2008), demonstrates substantial SNR improvements. The FPGA-based system achieves 41–47% higher spatial SNR and near-theoretical temporal SNR scaling, reaching a $4.98\times$ improvement over the commercial reference. These results highlight the effectiveness of hardware-level synchronization for improving the practical recoverability of high-frequency spatial detail under reduced acquisition time. The modular architecture is applicable to a broad range of point-scanning instruments beyond electron microscopy.

## Introduction

Precision scan-signal generation and synchronous high-fidelity data acquisition constitute the enabling foundation for a broad class of scientific imaging and nondestructive evaluation (NDE) instruments (Minsky 1961; Binnig et al. 1982; Huang et al. 1991; Choi et al. 2024; Gregorat et al. 2024). Such scan–synchronization technology is employed extensively in high-resolution imaging platforms, including confocal microscopes and electron microscopes (Pawley 2006; Wilson and Sheppard 1984; Davidovits and Egger 1969), while also governing defect-detection sensitivity and spatial resolution in ultrasonic and eddy-current NDE systems (Hassani and Dackermann 2023; Santos et al. 2023). The growing demand for programmable scan waveforms and real-time signal processing has motivated the development of field-programmable gate array (FPGA)-based integrated platforms capable of providing both deterministic hardware-level synchronization and flexible software-configurable parameter control (Choi et al. 2024; Gregorat et al. 2024).

Pioneering work on confocal microscopy (Minsky 1961), atomic force microscopy (AFM) (Binnig et al. 1982), and optical coherence tomography (OCT) (Huang et al. 1991) demonstrated that hardware-centric point-scanning architectures are highly effective for improving spatial resolution and signal sensitivity. The point-scanning paradigm has since been adopted across spectroscopic analysis, ultrasonic testing, and numerous other metrology domains. Nevertheless, instrument-specific scan controllers and data acquisition circuits continue to be developed independently for each platform, underscoring the persistent need for a versatile, instrument-agnostic scan and data acquisition system.

Modern metrology is advancing along three converging directions: higher speed, higher dimensionality, and data-driven algorithmic fusion. In three-dimensional surface profilometry, chromatic confocal techniques have achieved sub-micrometer vertical resolution (Chen and Chen 2023). In fluorescence imaging, the integration of structured illumination microscopy (SIM) with fluorescence lifetime imaging microscopy (FLIM) has enabled depth-resolved real-time analysis of biological specimens (Gustafsson 2000; Park and Gao 2024). In spectral mapping, the combination of Raman imaging with machine-learning-based noise learning has yielded signal-to-noise ratio (SNR) improvements exceeding one order of magnitude (Qi et al. 2023; Zhou et al. 2025; He et al. 2024). These advances share a common prerequisite: high temporal-resolution scan trajectory control coupled with wideband synchronous acquisition, further motivating the development of a universal scan platform.

Within electron microscopy (EM), scan–synchronization technology is likewise undergoing rapid refinement. Four-dimensional scanning transmission electron microscopy (4D-STEM) and ptychography reconstruct atomic-scale phase and structural information by collecting two-dimensional diffraction patterns at each raster point, demanding precise raster timing control and pixel-level synchronization (Ophus 2019; Seifer 2023). Multi-beam SEM has achieved approximately two orders of magnitude improvement in throughput by parallelizing tens of electron beams (Eberle et al. 2015), while environmental SEM (ESEM) permits high-resolution observation of non-conductive specimens under elevated-pressure gaseous environments, thereby relaxing specimen preparation constraints (Danilatos 1988). All of these techniques rely fundamentally on precise beam trajectory control and real-time synchronized detection. In the context of large-area serial SEM imaging, flexible data acquisition architectures have enabled automated array tomography workflows with nanometer-scale resolution, further highlighting the importance of programmable scan systems (Kim et al. 2020). More recently, smart microscopy approaches that combine rapid image acquisition with predictive subregion selection have demonstrated significant reductions in total beam exposure time in connectomics applications, a capability that depends critically on the deterministic timing control provided by hardware-level implementations (Athey et al. 2025).

In this paper, we report the design and implementation of a scanning and data acquisition system that integrates raster scan waveform generation, hardware-synchronized DAC/ADC operation, and real-time USB data streaming within a single module based on the Xilinx Artix-7 (XC7A35T) FPGA. The system is designed to achieve programmable raster waveforms and clock-cycle-level deterministic synchronization on a low-cost FPGA platform. Circuit design and PCB fabrication section describes the analog front-end circuit design and printed circuit board (PCB) fabrication. FPGA firmware architecture and Artix-7 implementation section presents the modular FPGA firmware architecture and signal-processing pipeline. Integration with a commercial SEM and imaging results section reports the results of integration with a commercial SEM (ModuleSci PicoEye-100). Through this work, we demonstrate the feasibility of an FPGA-based universal scan platform applicable to diverse imaging and metrology instruments.

## Circuit design and PCB fabrication

### Hardware architecture

**Fig. 1:**
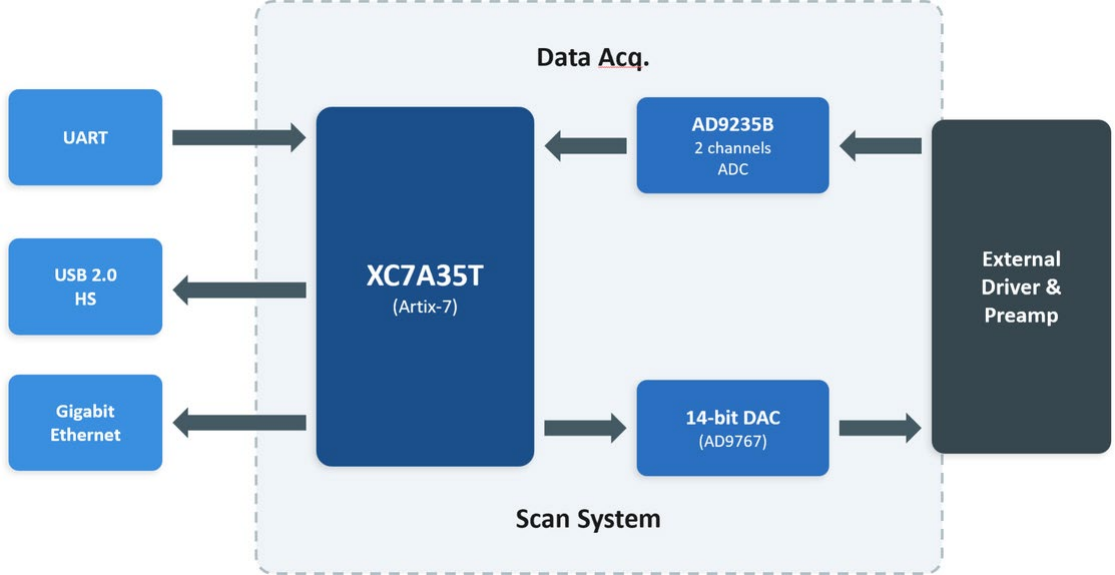
Overall architecture of the FPGA-based scanning and data acquisition platform built around the Xilinx Artix-7 (XC7A35T). The system comprises four functional blocks: a dual-channel ADC (AD9235) for SE/BSE signal acquisition, a dual-channel DAC (AD9767) for X–Y raster scan waveform generation, communication interfaces (USB 2.0 HS, Gigabit Ethernet, UART), and an external scan-coil driver stage

The proposed system comprises four functional blocks: a data acquisition unit (ADC), a scan control unit (DAC), communication interfaces, and an external driver stage. Figure 1 shows the overall system block diagram. The architecture is organized around the FPGA as a central hub; each block operates as a functionally independent module while sharing a common clock domain for real-time synchronization.

The data acquisition unit employs a dual-channel AD9235BRUZ-20 ADC (12-bit resolution, 20 MSPS maximum sampling rate). The two channels are allocated to the simultaneous acquisition of secondary electron (SE) and backscattered electron (BSE) signals; digitized sample data are transferred to the FPGA through a parallel bus interface. The scan control unit utilizes a dual-channel AD9767ASTZRL DAC (14-bit resolution, 125 MSPS maximum update rate). The X-axis and Y-axis raster scan waveforms synthesized within the FPGA are converted to analog voltages by the DAC and routed to external scan-coil driver amplifiers. This configuration enables both precision raster scan control and multi-channel signal acquisition to be realized within a single hardware module. The hardware architecture inherits the design philosophy of previously reported FPGA-based SEM scan generators and signal acquisition systems (Rahangdale et al. 2016; Peng et al. 2024), while physically consolidating the dual-channel ADC and DAC onto a single expansion board, thereby achieving simultaneous miniaturization and improved inter-channel synchronization.

The communication subsystem adopts an FT232HL-based USB 2.0 High-Speed interface as the primary data path, supporting a sustained throughput of up to 40 MB/s in Synchronous FIFO mode. A Gigabit Ethernet interface is additionally provisioned for remote control and network-based data management, and a UART interface operates in parallel for firmware debugging and low-speed command transfer. The FPGA (XC7A35T) serves as the central processing unit that orchestrates scan waveform synthesis, data acquisition timing control, and communication protocol management within a single device.

### PCB design and fabrication

The schematic and layout of the ADC/DAC expansion board were designed using EasyEDA Professional. The board dimensions are 145 mm $\times$ 100 mm with a four-layer stack-up. During layout, the analog conversion section and the digital control section were physically partitioned to minimize digital switching noise coupling into the analog signal path. The ADC input stage incorporates an anti-aliasing low-pass filter and signal conditioning circuitry to ensure stable sampling conditions satisfying the Nyquist criterion. The DAC output stage includes a current-to-voltage conversion circuit and a reconstruction filter to suppress harmonic content and preserve output waveform fidelity.

The power supply subsystem converts externally supplied $-3.3$ V and $+15$ V inputs to regulated $+5$ V and $+3.3$ V rails through on-board linear regulators. The analog and digital power rails are physically separated, and 0.1 $\mu$F and 10 $\mu$F bypass capacitors are placed in parallel at each IC power pin to improve transient response and suppress supply noise. For the high-speed signal paths, the ADC/DAC–FPGA data lines are routed with a 100 $\Omega$ differential impedance target, and the clock distribution lines are routed with a 50 $\Omega$ single-ended impedance target, thereby minimizing jitter and ground bounce.

### Assembly and inspection

Following PCB fabrication, surface-mount technology (SMT) components were assembled using solder paste stencil printing, automated pick-and-place, and reflow soldering with a temperature profile optimized for lead-free solder. Through-hole components, including connectors and bulk capacitors, were assembled by wave soldering. All manufacturing and assembly processes were carried out by JLCPCB.

**Fig. 2:**
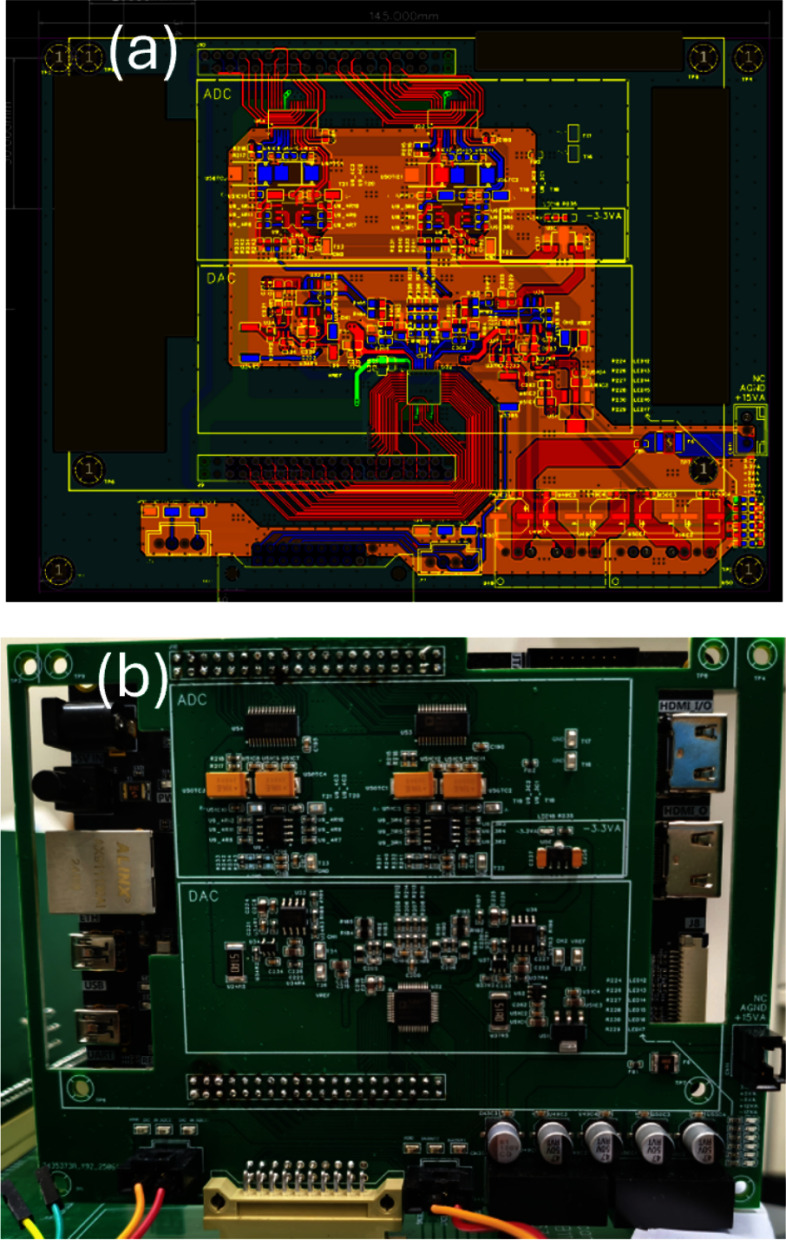
Implementation of the ADC/DAC expansion board: (**a**) four-layer PCB artwork (145 mm $\times$ 100 mm) designed in EasyEDA Professional, showing the physical separation of analog and digital domains; (**b**) photograph of the assembled board mounted on the AX7035B FPGA development board via a 40-pin expansion connector

The completed ADC/DAC expansion board connects directly to the AX7035B FPGA development board through a 40-pin expansion connector. This connector carries the 12-bit dual-channel ADC data bus, the 14-bit dual-channel DAC output bus, and clock and control signals, all of which are mapped one-to-one to the FPGA I/O pins. This architecture ensures that scan waveform generation and detector signal acquisition execute synchronously within the same clock domain. Figure 2 shows the completed expansion board and its connection to the FPGA development board.

## FPGA firmware architecture and Artix-7 implementation

**Fig. 3:**
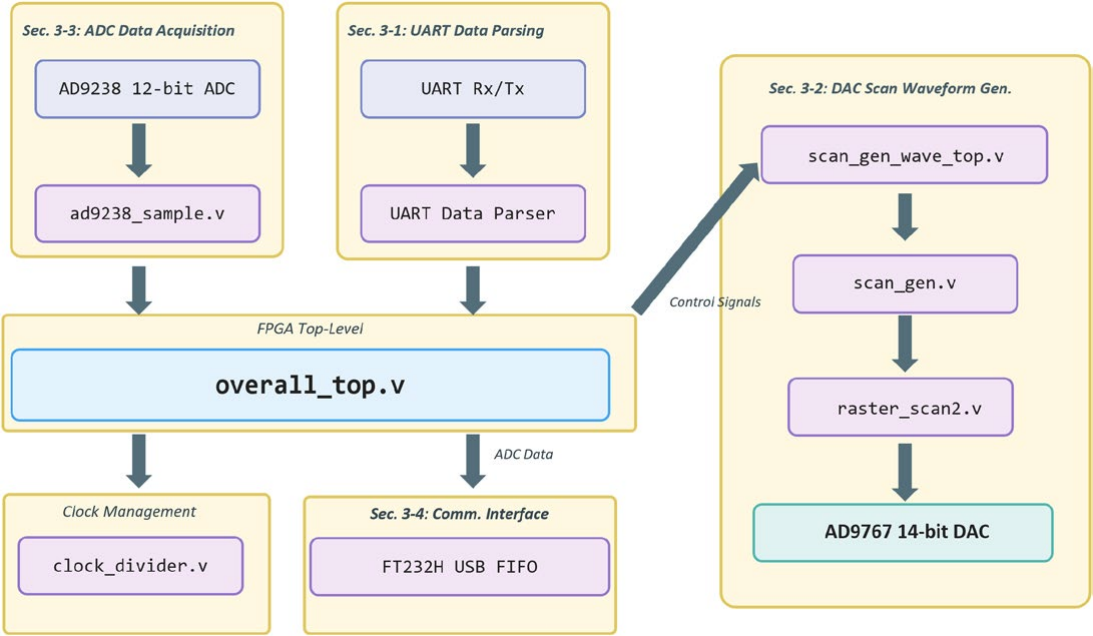
Block diagram of the FPGA firmware architecture. The top-level module (overall_top.v) integrates four major functional blocks: UART-based command parser, raster scan waveform generator (ScanGen), dual-channel ADC sampling engine, and USB/Ethernet communication controller. Arrows indicate the data flow and control signal paths among the modules

The FPGA firmware is organized into four core functions—command parsing, scan waveform generation, data acquisition, and communication—each implemented as an independent Verilog HDL module and hierarchically integrated within a top-level design file (overall_top.v) (Gregorat et al. 2024). Figure 3 illustrates the data flow and signal interconnections among the UART command parser, ADC sampling engine, DAC scan waveform generator, and communication modules.

### Command parsing module

Serial data received from the host PC via the UART interface are interpreted by the uart_test_data_parser.v and data_parser.v modules within the FPGA. The incoming byte stream is analyzed according to a fixed-length packet protocol comprising a header, command code, parameter field, and terminator. Only frames that pass integrity verification are committed to the internal register file, enabling key operational parameters—scan resolution, oversampling factor, scan rate, and scan mode—to be dynamically reconfigured during an ongoing experiment without halting the system. The UART $\rightarrow$ Parser $\rightarrow$ Top-Level path in Fig. 3 illustrates this command delivery and parameter update flow.

### Scan waveform generation module

Raster scan waveform synthesis is performed through the coordinated operation of the scan_gen.v, raster_scan2.v, and scan_gen_wave_top.v modules (Rahangdale et al. 2016). A sawtooth waveform corresponding to the fast-scan (X) axis and a staircase waveform corresponding to the slow-scan (Y) axis are generated independently; their combination defines a two-dimensional raster trajectory. The synthesized 14-bit digital waveform data are transferred to the DAC (AD9767) through a parallel register interface, converted to analog voltages, and applied to the external scan-coil driver amplifier.

The waveform generator adopts a parameterized design methodology, supporting scan resolutions ranging from $512 \times 512$ to $4{,}096 \times 4{,}096$ pixels. Extension of the internal register bit width and address space permits scaling up to $16{,}384 \times 16{,}384$ scan points. Combined with the 14-bit DAC resolution, this design enables the simultaneous realization of a wide scan field of view and high spatial resolution. The ScanGen-to-DAC output path in Fig. 3 depicts this data flow.

### Data acquisition module

Detector signal digitization is performed by the ad9238_sample.v module. Dual 12-bit sample data from the external ADC (AD9235/AD9238) are latched into the FPGA in synchronization with a stable sampling clock generated by the clock_divider.v module (Choi et al. 2024). Acquired data are buffered in a first-in first-out (FIFO) memory and forwarded to higher-level logic for subsequent communication processing. The dual-channel architecture supports simultaneous SE and BSE signal acquisition; inter-channel timing skew is minimized through the common clock domain design.

The acquired dual-channel data undergo real-time averaging and FIFO-based interleaving for multiplexed transmission. Specifically, odd-indexed samples are mapped to channel 1 and even-indexed samples to channel 2, merging both channels into a single data stream while preserving signal integrity under continuous streaming conditions. The ADC $\rightarrow$ Sampling $\rightarrow$ Top-Level path in Fig. 3 depicts this data flow.

### Communication module

High-speed data transfer is implemented via the FT232H-based USB 2.0 High-Speed Synchronous FIFO mode, which provides a sustained throughput of up to 40 MB/s and enables real-time streaming of frames at $4{,}096 \times 4{,}096$ resolution. To accommodate future scalability, the hardware and firmware architectures are designed to support the addition of a Gigabit Ethernet interface. The UART interface is retained for debugging and low-speed control, operating independently in parallel with the high-speed streaming path. This multi-interface architecture permits seamless switching between local standalone operation and remote network-based operation as dictated by experimental requirements.

### Scan waveform verification

To verify the operation of the scan waveform generation module, the custom-designed ADC/DAC expansion board was mounted on the Artix-7 FPGA (XC7A35T) development board, and the DAC output waveforms were recorded using a RIGOL HDO4204 digital oscilloscope (200 MHz bandwidth, 12-bit vertical resolution). The register settings applied for the verification experiment are summarized in Table 1.

**Table 1:** Test register parameters for scan waveform generation

Parameter	Value	Description
reg_Npara	25	Number of averaging cycles per pixel
reg_Startx	0	X-axis scan start address
reg_Starty	0	Y-axis scan start address
reg_Endx	4095	X-axis scan end address (12-bit full scale)
reg_Endy	4095	Y-axis scan end address (12-bit full scale)
reg_Stepsizeup	2	Forward step size exponent ($2^n$)
reg_Stepsizedown	8	Retrace step size exponent ($2^n$)
reg_Cont_or_not	1	Continuous scan mode enable
reg_endstring	0	Scan termination flag

**Fig. 4:**
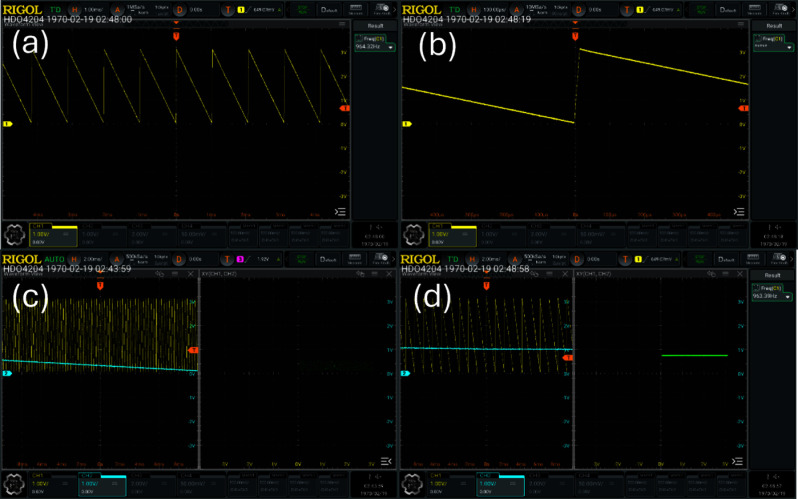
Oscilloscope measurements of DAC-generated scanning waveforms: (**a**) inverted X-axis sawtooth waveform at the output of the external driver amplifier, with a measured fundamental frequency of approximately 961 Hz; (**b**) magnified view of the flyback (retrace) region, showing the fast retrace enabled by reg_Stepsizedown $= 8$; (**c**, **d**) Lissajous (X–Y) display confirming stable phase alignment between the fast-scan and slow-scan axes

Figure 4a shows the inverted X-axis sawtooth waveform measured at the output of the external driver amplifier; the measured fundamental frequency of approximately 961 Hz is in agreement with the theoretical line-scan rate calculated from the configured scan parameters. Figure 4b presents a magnified view of the flyback region, demonstrating the fast retrace behavior enabled by the reg_Stepsizedown $= 8$ setting. This programmable retrace speed reduces the fractional time overhead of the flyback interval, thereby improving the overall frame acquisition throughput. Figure 4c and d show the X–Y scan signals observed in Lissajous mode, confirming stable inter-axis phase alignment at the 961 Hz scan rate and verifying that the raster waveform is synthesized in accordance with the design intent.

### Data acquisition verification

The data acquisition subsystem was verified with the system clock set to 50 MHz. At each scan point, samples are accumulated over reg_Npara $\times$ 2 $=$ 50 clock cycles for real-time averaging, yielding an effective SNR enhancement. The averaged data are stored in FIFO memory and streamed in real time via odd/even index interleaving, where odd-indexed samples correspond to channel 1 and even-indexed samples to channel 2.

**Fig. 5:**
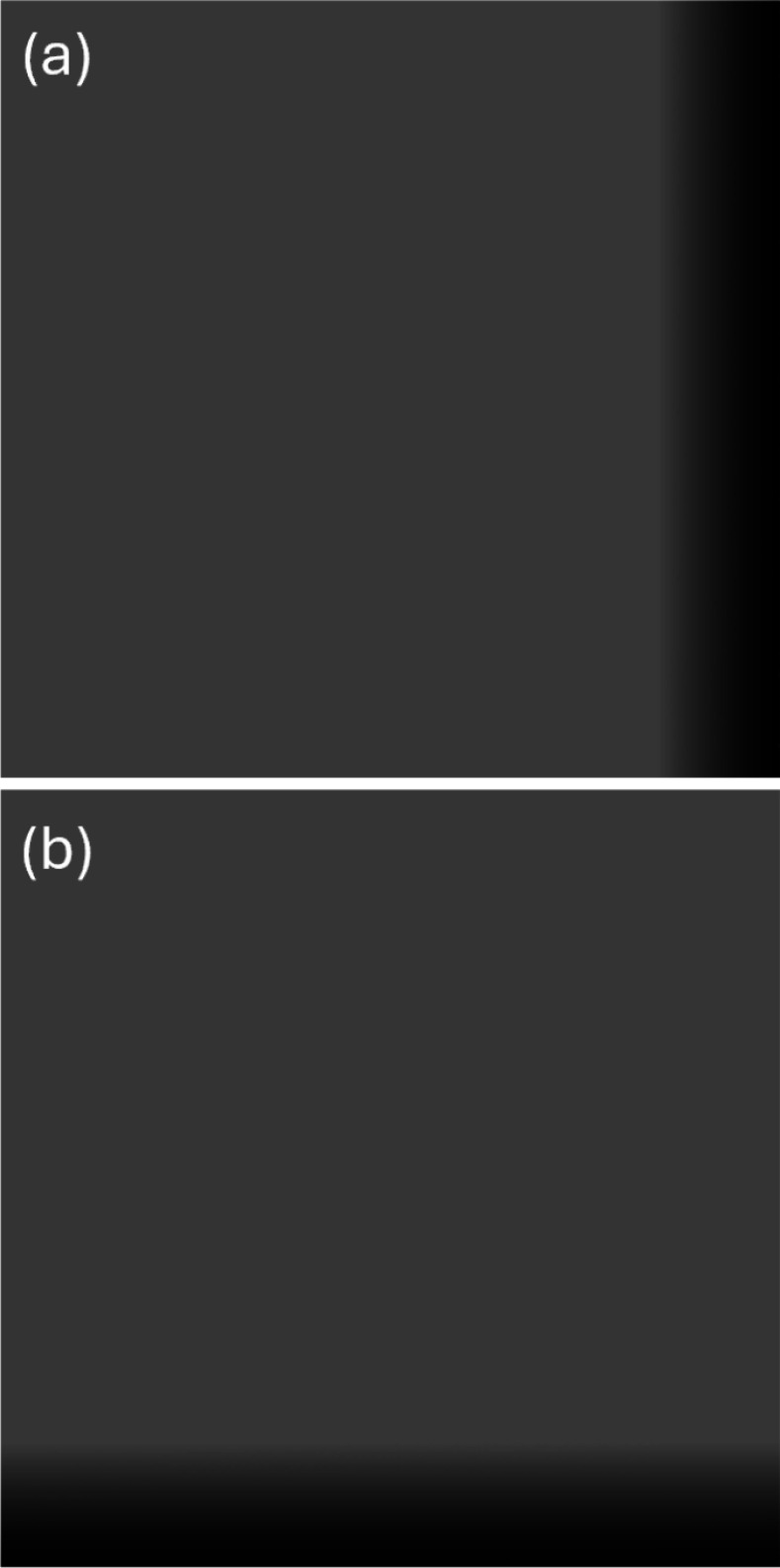
Verification of dual-channel ADC data acquisition via FIFO-based interleaving: (**a**) channel 1 output showing a linear ramp signal corresponding to the fast X-scan axis; (**b**) channel 2 output showing a staircase signal corresponding to the slow Y-scan axis. Both channels were acquired in full temporal synchronization

For the verification experiment, two distinct test waveforms were injected into channels 1 and 2. Figure 5a shows the linear ramp signal acquired on channel 1, corresponding to the fast X-scan axis, and Fig. 5b shows the staircase signal acquired on channel 2, corresponding to the slow Y-scan axis. Both channels were confirmed to be digitized in full temporal synchronization, demonstrating that the FPGA-based interleaving and synchronization logic operates as designed. This result verifies that temporal alignment between the scan waveform and the detector signal is maintained reliably through the dual-ADC architecture, establishing the foundation for real-time SEM image streaming described in the following section.

## Integration with a commercial SEM and imaging results

### System integration

The FPGA-based scanning and data acquisition module validated in FPGA firmware architecture and Artix-7 implementation section was integrated into a commercial SEM system (ModuleSci PicoEye-100). During integration, the DAC outputs of the FPGA module were connected directly to the SEM deflection electronics, and the ADC inputs were connected to the preamplifier output of the SE detector. This hardware interfacing established a closed-loop imaging configuration in which the DAC-generated raster scan waveform directly drives the electron beam deflection, while the SE signal arising from beam–specimen interaction is synchronously digitized in real time.

Because scan waveform generation, detector signal acquisition, and USB data transfer are all executed within a common clock domain on a single FPGA device, temporal alignment between the scan position and the detector signal is guaranteed at the hardware level. This confirms that the dual-channel synchronization performance verified with test signals in Data acquisition verification section remains valid under actual SEM operating conditions.

### Imaging results and analysis

**Fig. 6:**
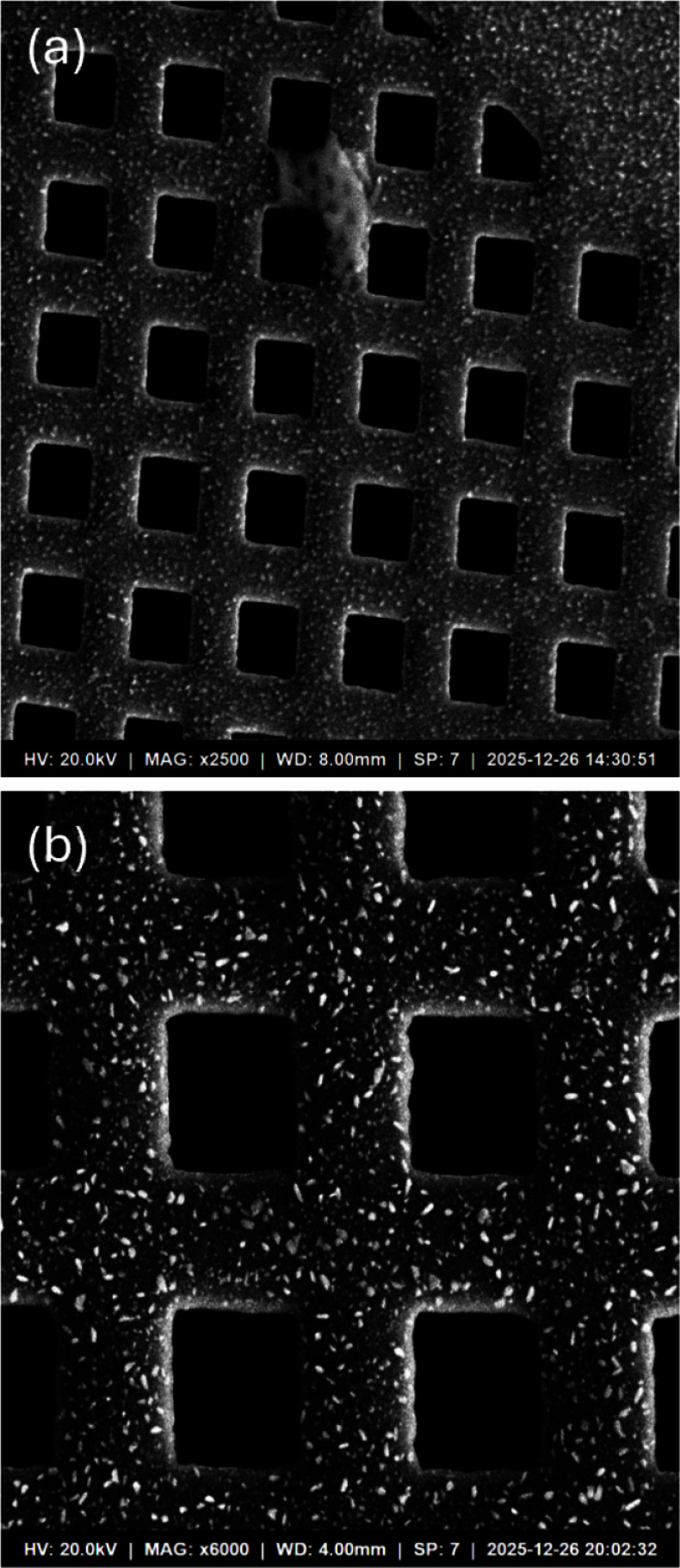
Secondary electron (SE) images of a calibration grid specimen with a pitch of 125 $\mu$m acquired using the FPGA-based scanning and data acquisition system integrated with the ModuleSci PicoEye-100 SEM. The original scan resolution was $1024 \times 1024$ pixels; however, only the effective imaging area of $880 \times 880$ pixels is displayed to exclude the flyback region. **a** Image acquired at a magnification of $\times 2500$. **b** Image acquired at a magnification of $\times 6000$. The periodic grid pattern is clearly resolved in both images, demonstrating stable raster scanning and synchronized data acquisition

To verify the imaging performance of the integrated system in the fast-scan mode that is routinely used for alignment, focusing and field-of-view selection in SEM operation, SE-mode images were acquired from a standard calibration grid specimen with a pitch of 125 $\mu$m. The scan resolution was set to $1{,}024 \times 1{,}024$ pixels, and the total frame acquisition time was approximately 1 s. It should be emphasized that the imaging shown in this section represents the fast-scan/alignment regime that precedes a measurement; high-quality data acquisition for the quantitative image-quality benchmarking against the instrument’s built-in commercial acquisition channel was instead carried out at a longer per-frame acquisition time of 10 s, and is presented separately in Image quality characterization section. Because the flyback region of the sawtooth waveform does not contribute valid image data, only the effective imaging area of $880 \times 880$ pixels was extracted and used for analysis. Figure 6a and b present representative SEM images acquired at magnifications of $\times 2500$ and $\times 6000$, respectively.

The periodic grid pattern with a 125 $\mu$m pitch is clearly resolved in both images, and the edge contrast at the grid boundaries is well defined, confirming that the SE detection signal was synchronously acquired and correctly mapped to each pixel location within the effective field of view. This result demonstrates that the FPGA-based raster scan logic stably generates the two-dimensional scanning trajectory across the fast (X) and slow (Y) axes and acquires the detector response at each scan point without data loss.

Mild geometric distortion is observable near the image boundaries. This artifact is attributed to the transient settling behavior of the scan-coil driver amplifier during the flyback interval of the sawtooth waveform. As discussed in Scan waveform verification section, the flyback retrace speed is programmable through the reg_Stepsizedown parameter, and optimization of this parameter is expected to reduce the settling time and the associated image distortion. It should be noted that the present images were acquired without X–Y orthogonality correction or scale calibration. The application of software-based geometric correction algorithms in future work is anticipated to further improve the spatial accuracy and metrological precision of the acquired images.

## Image quality characterization

### Measurement protocol

Having demonstrated stable raster scanning and synchronized SE acquisition in the fast-scan/alignment regime in Integration with a commercial SEM and imaging results section, we now turn to the standard data-acquisition regime and present a quantitative image-quality comparison between the FPGA-based acquisition system and the instrument’s built-in commercial acquisition channel. All benchmarking measurements reported in this section were performed at a per-frame acquisition time of 10 s, which is the typical setting used in routine SEM data acquisition on this instrument. This is distinct from the fast-scan/alignment condition (approximately 1 s/frame) used for the imaging shown in Integration with a commercial SEM and imaging results section. Two metrics are assessed: signal-to-noise ratio (SNR) and spatial resolution.

#### SEM operating conditions

All comparison images were acquired on the same ModuleSci PicoEye-100 SEM under identical column conditions: an accelerating voltage of 20.0 kV, a working distance of 8.0 mm, and a spot-size setting of 7. The benchmark dataset for SNR and MTF analysis was acquired at a magnification of $\times$10,000 on a copper calibration mesh with a pitch of 125 $\mu$m, while the proof-of-concept images shown in Integration with a commercial SEM and imaging results section were acquired at $\times$2,500 and $\times$6,000 on the same specimen. For the FPGA-based system, the per-frame acquisition time was 10 s and frame averaging was performed offline over $N = 1$, 4, 9, 16 and 25 frames. The commercial reference acquired a single 30 s frame at a slower scan speed for the same field of view. Apart from the acquisition electronics under test, all electron-optical settings (high voltage, working distance, spot size, condenser/objective lens excitations) were held constant between the two systems, so that the only variable in the comparison is the acquisition path itself.

#### Processing pipeline

Because the two systems produce images in different formats (TIFF for the FPGA-based path and JPEG for the commercial reference), an identical four-step processing pipeline is applied to both datasets prior to any quantitative comparison.

*Step 1 – Grid-hole masking.* A binary mask is generated from the calibration-grid images so that pixels falling on the vacuum (hole) regions of the mesh apertures are excluded from all subsequent statistical analysis. This step removes the contribution of the dark hole regions, which would otherwise dominate the standard deviation in the bright (specimen) region and bias the SNR estimate.

*Step 2 – Histogram normalization.* Because raw 12-bit TIFF and JPEG-compressed outputs differ in dynamic range and noise floor, the intensity histograms over the unmasked (bright) regions are matched between the two systems prior to comparison. This step removes the systematic noise-floor bias introduced by JPEG compression so that the remaining differences reflect genuine acquisition-path performance rather than file-format artefacts.

*Step 3 – Sub-pixel drift correction.* For temporal SNR analysis, residual mechanical and thermal drift between successive frames is corrected by sub-pixel cross-correlation registration following the method of Guizar-Sicairos et al. (2008). This step is essential because uncorrected drift between frames degrades the effective averaging gain and prevents the temporal SNR from reaching its theoretical $\sqrt{N}$ scaling.

*Step 4 – Frame averaging.* After alignment, *N* registered frames are averaged pixel-by-pixel. Spatial SNR is then computed from the bright-region statistics of each averaged image, and temporal SNR is computed as the pixel-wise mean-to-standard-deviation ratio across the *N*-frame stack.

The same four-step pipeline was applied identically to the FPGA-based and the commercial datasets, ensuring a fair head-to-head comparison. Spatial SNR is defined as $\mu (I_{\textrm{bright}})/\sigma (I_{\textrm{bright}})$ and temporal SNR as the pixel-wise mean-to-standard-deviation ratio across groups of *N*-frame averages. For resolution, edge-spread function (ESF) and line-spread function (LSF) analyses are applied to vertical grid edges, and the FWHM and MTF50 are extracted after correcting for a $1.66\times$ pixel-size difference between the two systems identified from their embedded scale bars. It should be noted that the intrinsic FWHM and MTF of the imaging system are governed primarily by the electron-optical column of the SEM and are independent of the acquisition electronics; the primary contribution of the FPGA-based path is therefore its SNR performance and the corresponding more reliable practical recovery of high-frequency spatial detail.

**Fig. 7:**
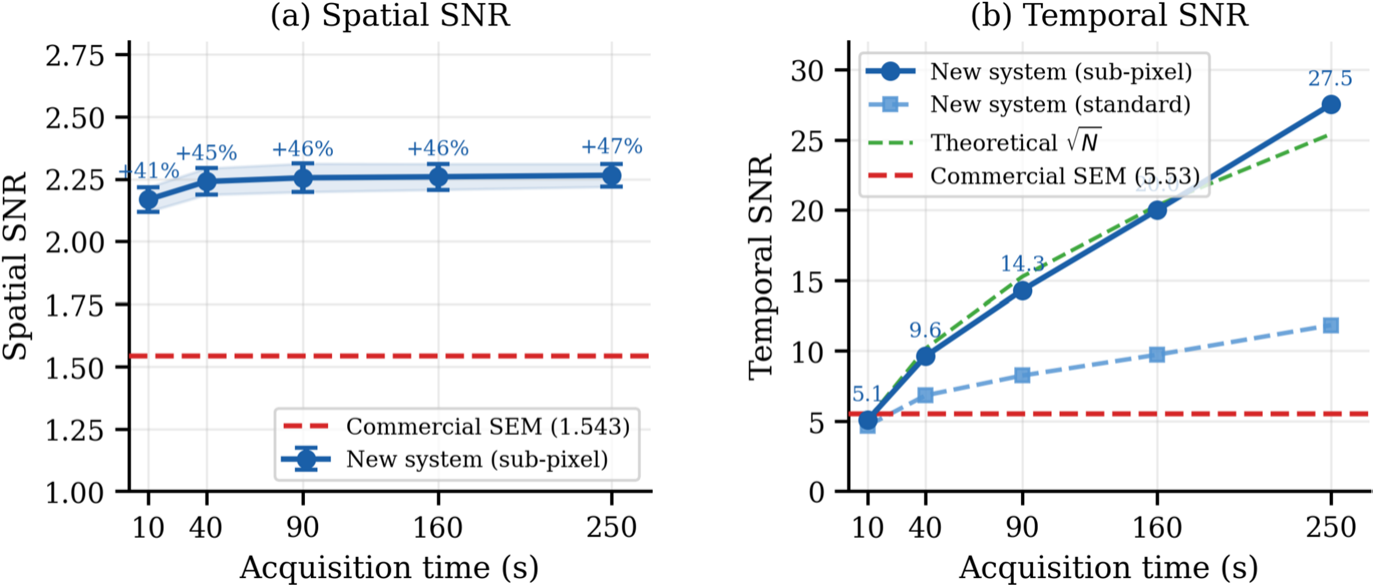
SNR comparison between the FPGA-based acquisition system and the commercial reference. **a** Spatial SNR vs. acquisition time. **b** Temporal SNR vs. acquisition time. Sub-pixel drift correction (filled circles) yields near-theoretical $\sqrt{N}$ scaling ($R^{2}=0.998$); standard alignment (open squares) achieves only $\approx$43% of the theoretical gain. Dashed red line: commercial reference

**Fig. 8:**
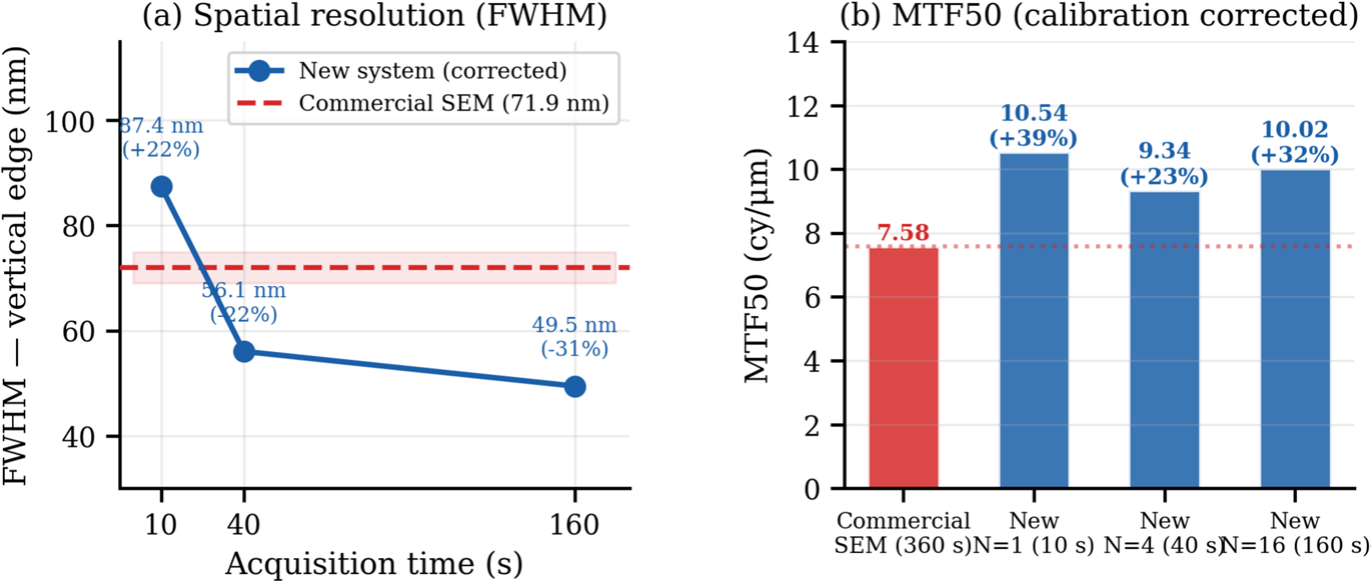
Calibration-corrected spatial resolution (vertical edge, $\times$10,000 magnification). **a** FWHM vs. acquisition time; dashed line: commercial reference (71.9 nm). **b** MTF50 after calibration correction; dotted line: commercial reference (7.58 cy/$\mu$m)

### Results

Figure 7 demonstrates that the FPGA-based acquisition system consistently outperforms the commercial reference in both spatial and temporal SNR across all tested conditions. In terms of spatial SNR, the FPGA system achieves an improvement of 41–47% compared to the commercial system, despite operating at one-third of the per-frame exposure time (10 s vs. 30 s). This indicates that the proposed architecture enables more efficient signal accumulation within a significantly reduced acquisition time. For temporal SNR, sub-pixel drift correction enables near-theoretical $\sqrt{N}$ scaling ($R^{2}=0.998$), reaching a value of 27.5 at $N=25$. This corresponds to a $4.98\times$ improvement over the commercial reference, which exhibits substantially lower scaling efficiency due to residual misalignment.

Figure 8 presents the calibration-corrected spatial resolution results. From $N \ge 4$, the FPGA system achieves both smaller FWHM (56.1 nm, −22%) and higher MTF50 (9.3–10.5 cy/$\mu$m, +23–39%) than the commercial reference. Because the intrinsic electron-optical resolution is governed by the SEM column itself and is independent of the acquisition electronics, these gains should be interpreted not as a change in the SEM’s intrinsic resolution but as a more reliable practical recovery of the high-frequency spatial detail already accessible to the column, enabled by the higher SNR and stable averaging of the proposed acquisition path. Table 2 summarizes the numerical comparison.

**Table 2:** Image quality comparison: FPGA-based system (sub-pixel alignment) vs. commercial reference

System/Cond.	*N*	Sp. SNR	Tmp. SNR	FWHM (nm)	MTF50 (cy/$\mu$m)
Commercial (30 s)	1	1.54	5.53	71.9	7.58
FPGA (10 s)	1	2.17	5.1	87.4	10.54
FPGA (40 s)	4	2.24	9.6	56.1	9.34
FPGA (90 s)	9	2.26	14.3	—	—
FPGA (160 s)	16	2.26	20.0	49.5	10.02
FPGA (250 s)	25	2.27	27.5	—	—

## Discussion

The results demonstrate that the proposed FPGA-based acquisition system provides consistent improvements in SNR compared to the commercial reference, while operating at a substantially reduced per-frame exposure time.

The enhancement in spatial SNR (41–47%) indicates more efficient signal accumulation within the proposed architecture. This can be attributed to deterministic synchronization between scan waveform generation and detector signal acquisition within a unified FPGA clock domain, which minimizes timing jitter and preserves signal integrity.

The near-theoretical $\sqrt{N}$ scaling of temporal SNR ($R^{2}=0.998$) further confirms that signal coherence is effectively maintained during frame averaging. The marked reduction in performance under standard alignment ($\approx$43% efficiency) highlights the critical role of sub-pixel drift correction in achieving optimal averaging performance.

Because the intrinsic electron-optical resolution is set by the SEM column itself and is independent of the acquisition electronics, the observed reductions in FWHM and corresponding gains in MTF50 should be interpreted not as a change of the SEM’s optical resolution, but as a more reliable practical recovery of the high-frequency spatial information already accessible to the column, enabled by the higher SNR and stable averaging of the proposed acquisition path. This indicates that acquisition electronics can have a non-negligible impact on the effective image quality delivered to the user.

Compared to conventional SEM acquisition systems with fixed processing pipelines, the proposed FPGA-based architecture offers greater flexibility and precise timing control. This is particularly advantageous in applications requiring reduced exposure time or high-speed imaging, where maintaining SNR is critical.

Nevertheless, several limitations should be noted. Geometric distortion near image boundaries indicates that scan linearity and orthogonality correction are not yet fully implemented. In addition, validation has been limited to a single SEM platform. Further studies are required to evaluate performance across different instruments and imaging conditions.

Future work will focus on real-time geometric correction, multi-channel acquisition, and higher-throughput data interfaces. These developments are expected to further improve system performance and expand its applicability to a wider range of point-scanning imaging modalities.

Overall, the results demonstrate that hardware-level synchronization within an FPGA-based architecture can significantly improve signal fidelity and acquisition efficiency in SEM imaging.

## Conclusions

We have designed and validated an FPGA-based scanning and data acquisition system for SEM, integrating raster scan waveform generation, dual-channel ADC/DAC synchronization, and high-speed USB data streaming on a single Artix-7 (XC7A35T) module. Oscilloscope tests confirmed correct waveform generation, and integration with the ModuleSci PicoEye-100 SEM produced clear SE images of a calibration grid in the fast-scan mode (approximately 1 s per frame) used for alignment and focusing, while the quantitative benchmarking against the built-in commercial acquisition channel was carried out under standard data-acquisition conditions at a per-frame acquisition time of 10 s.

Quantitative image-quality benchmarking demonstrated that the FPGA-based system outperforms the instrument’s built-in commercial acquisition channel in SNR across all tested conditions, with spatial SNR improved by 41–47% and temporal SNR scaling near-theoretically with frame averaging when sub-pixel drift correction is applied. Although the intrinsic electron-optical resolution of the SEM is governed by the column itself and is not altered by the acquisition electronics, the higher SNR and stable averaging of the proposed path also yield smaller FWHM and higher MTF50, reflecting an improvement in the practical recoverability of high-frequency spatial detail rather than in the intrinsic resolution.

These results indicate that hardware-level synchronization within a unified FPGA architecture can significantly enhance signal fidelity and acquisition efficiency in SEM imaging. Future work will address geometric calibration, remote operation via Gigabit Ethernet, and concurrent multi-channel acquisition. The proposed architecture provides a scalable foundation for next-generation programmable scanning and data acquisition systems.

## Data Availability

Not applicable.
